# 

**Published:** 1898-07

**Authors:** 


### CONTENTS.

ORIGINAL COMMUNICATIONS—

MALIGNANT DISEASE OF THE UTERUS. By Louis Frank, M.D., Louisville, Ky........ 289

EARLY EXPERIENCES AND REMINISCENCES OF FORTY YEARS OF PRACTICE. By G. G. Roy, M.D., Atlanta, Ga........ 293

PERITONSILLAR ABSCESS. By Dunbar Roy, A.B., M.D., Atlanta, Ga........ 296

MENTAL DISTURBANCES IN TURBINATE HYPERTROPHIES, OR NASAL STENOSIS. By John F. Woodward, M.D., Norfolk, Va........ 306

INDICATIONS FOR AND ASEPTIC TECHNIQUE OF UTERINE DRAINAGE AFTER LABOR AND ABORTION. By R. R. Kime, M.D., Atlanta, Ga........ 313

EDITORIAL—

THE DENVER MEETING........ 322

THE ATLANTA COLLEGE OF PHYSICIANS AND SURGEONS........ 325

MEDICAL ITEMS........ 327

BOOK REVIEWS........ 334

SELECTIONS AND ABSTRACTS........ 339

MEDICAL ITEMS—CONTINUED........ x, xi, xii, xiii, xiv, xvi, xvii

ADVERTISERS' INDEX TO ADVERTISEMENTS........ xviii

CLASSIFIED INDEX TO ADVERTISEMENTS........ xix

